# Weakened Effective Connectivity Related to Electroacupuncture in Stroke Patients with Prolonged Flaccid Paralysis: An EEG Pilot Study

**DOI:** 10.1155/2021/6641506

**Published:** 2021-03-09

**Authors:** Yi-Fang Lin, Xin-Hua Liu, Zheng-Yu Cui, Zuo-Ting Song, Fei Zou, Shu-Geng Chen, Xiao-Yang Kang, Bin Ye, Qiang Wang, Jing Tian, Jie Jia

**Affiliations:** ^1^Department of Rehabilitation Medicine, Huashan Hospital, Fudan University, Shanghai, China; ^2^Engineering Research Center of AI & Robotics, Ministry of Education, Shanghai Engineering Research Center of AI & Robotics, MOE Frontiers Center for Brain Science, Institute of AI and Robotics, Academy for Engineering & Technology, Fudan University, Shanghai, China; ^3^Department of Rehabilitation Medicine, The Shanghai Third Rehabilitation Hospital, Shanghai, China; ^4^Department of Rehabilitation Medicine, Shanghai Jing'an District Central Hospital, Shanghai, China

## Abstract

Flaccid paralysis in the upper extremity is a severe motor impairment after stroke, which exists for weeks, months, or even years. Electroacupuncture treatment is one of the most widely used TCM therapeutic interventions for poststroke flaccid paralysis. However, the response to electroacupuncture in different durations of flaccid stage poststroke as well as in the topological configuration of the cortical network remains unclear. The objectives of this study are to explore the disruption of the cortical network in patients in different durations of flaccid stage and observe dynamic network reorganization during and after electroacupuncture. Resting-state networks were constructed from 18 subjects with flaccid upper extremity by partial directed coherence (PDC) analysis of multichannel EEG. They were allocated to three groups according to time after flaccid paralysis: the short-duration group (those with flaccidity for less than two months), the medium-duration group (those with flaccidity between two months and six months), and the long-duration group (those with flaccidity over six months). Compared with short-duration flaccid subjects, weakened effective connectivity was presented in medium-duration and long-duration groups before electroacupuncture. The long-duration group has no response in the cortical network during electroacupuncture. The global network measures of EEG data (sPDC, mPDC, and *N*) indicated that there was no significant difference among the three groups. These results suggested that the network connectivity reduced and weakly responded to electroacupuncture in patients with flaccid paralysis for over six months. These findings may help us to modulate the formulation of electroacupuncture treatment according to different durations of the flaccid upper extremity.

## 1. Introduction

Flaccid paralysis is the most severe motor impairment following stroke, characterized by weakness and reduced muscle tone [1]. According to the Brunnstrom recovery stage, stroke survivors go through from initial flaccid paralysis to a number of stages characterized by spasticity, to the development of selective control of movement, and finally to the restoration of normal movement [2]. The time required for patients to go through from one stage to the next varies. The time required from flaccid phase to spasticity occurrence in stroke patients is one to three weeks in general [3]. Previous research has suggested that the prevalence of spasticity at 2-10 days, 2 weeks, and 4 weeks since stroke onset is 4%, 20.2%-24.5%, and 27%, respectively [4]. However, the duration of flaccid stage lasted for months, or even for years in some individuals with stroke. As we know, independence by activities of daily living (ADL) relies to a great extent on the motor function recovery of the upper extremity [5]. Prolonged flaccid upper extremity impacts independence and accelerates disability. Prolonged flaccidity has been defined as muscle hypotonia lasting for more than 2 months after stroke [6]. If a patient has flaccid paralysis for more than one year since stroke onset, his motor recovery occurs later or proceeds more slowly and he may not be able to regain any function from treatment [6, 7].

As an effective nondrug treatment, electroacupuncture has been widely used poststroke for upper extremity recovery. Several studies have reported that Quchi (LI11), Shousanli (LI10), and Hegu (LI4) are the frequently used and effective acupoints of motor recovery in patients with stroke [8–10]. These acupoints belong to the large intestine meridians. Stimulating them can promote the restoration of free flow of essence and blood according to the TCM theory [11]. A large number of studies published in recent years showed that electroacupuncture used for poststroke upper extremity rehabilitation was a growing field of interest. However, these researches paid little attention to flaccid upper extremity. Little attention was paid to the cortical impact (e.g., neuroplasticity, functional connectivity, cortical network) aroused by electroacupuncture in this population. Neuroplasticity refers to the capacity of the nervous system to modify its structure and function in response to environmental changes, which is strongly related to brain remodeling [12]. Promotion and modulation of neuroplasticity through therapeutic techniques (e.g., noninvasive brain stimulation, acupuncture/electroacupuncture, task-oriented training) are the keys to facilitate motor recovery [13, 14]. Thus, investigating the electroacupuncture-related brain activity among patients with different durations of flaccid stage is essential. It may have important implications for adjusting the electroacupuncture prescription in clinical practice.

EEG is convenient to detect real-time cortical activity as a high-time-resolution electrophysiological technique [15]. It is used widely to detect the brain oscillations, cortex excitability, cortical network, and electrophysiological biomarker in multiple disciplines through various EEG signal processing and feature extraction (e.g., spectral analysis, time-frequency analysis, connectivity analysis) [16–21]. Given that brain activity is a dynamic and complex network with each area's communication and coordination, effective connectivity is selected to investigate the different brain area connectivity and plasticity in this study. Granger causality-based effective connectivity is regarded as an important dimension of functional connectivity under specific conditions, which estimated the causal influence from one neural region to another [22]. A convenient measure of effective cortical connectivity is partial directed coherence (PDC) which is often used to deduce the intensity of information flow over the brain from EEG data [23, 24].

Brain oscillations of twenty-three flaccid stroke patients have been investigated by spectral analysis in our preliminary study [25]. We found that beta-band oscillations increased and delta-band oscillations decreased in the motor cortex in patients with flaccid upper extremity during electroacupuncture [25]. However, neural oscillations based on spectral analysis are not enough to reveal the electroacupuncture-related brain activity in patients with flaccidity. Different durations of flaccid stage were ignored in our previous research. Hence, it is necessary to implement further investigation and in-depth cortical network analyses.

With these considerations, based on our preliminary study, we continuously enrolled patients with flaccid upper extremity after stroke and constructed effective cortical networks using PDC analysis of 32-channel EEG signals. Connective deviation of patients was uncovered by comparing with three different durations of flaccid stage groups from peculiarities of global network connectivity and individual connection. We hypothesize that the cortical connectivity descended and the response to electroacupuncture weakened along with the prolonged duration of flaccid stage.

## 2. Methods

### 2.1. Study Design and Participants

**Figure 1:**
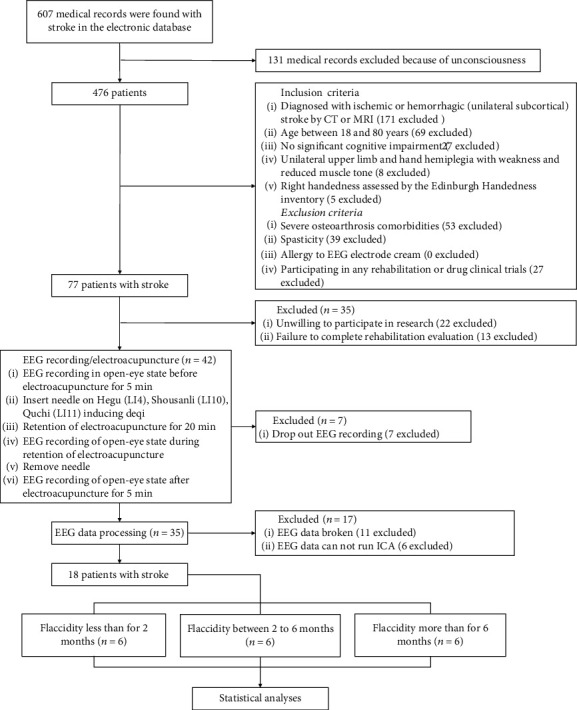
Flow chart of the study participant.

Eighteen patients with flaccid upper extremity after stroke were recruited from the Department of Rehabilitation Medicine, Huashan Hospital, and Shanghai Third Rehabilitation Hospital. The types of stroke and lesions were confirmed by a clinical CT or MRI scanner. The inclusion criteria were (1) adult patients with first-time stroke; (2) Mini-Mental State Examination ≥ 23; (3) unilateral upper limb and hand hemiplegia with weakness and reduced muscle tone; and (4) right-handedness assessed by the Edinburgh Handedness Inventory [26]. Patients were excluded if they had (1) spasticity (Modified Ashworth Spasticity Scale ≥ 1) of the affected arm; (2) severe osteoarthrosis comorbidities; (3) allergy to EEG electrode cream; and (4) participating in other rehabilitation or drug clinical trials. Eighteen patients were allocated to three groups according to their duration of flaccid stage: the short-duration group (SDG, *n* = 6, 4 males and 2 female), in which patients' flaccid stage was less than for two months; the medium-duration group (MDG, *n* = 6, 5 males and 1 female), in which patients' flaccid stage was between two months and six months; and the long-duration group (LDG, *n* = 6, 4 males and 2 female), in which patients' flaccid stage was over six months. All patients received the electroacupuncture treatment once and EEG recording. The flowchart of participants through the study is shown in Figure 1. All patients signed informed consent forms before the participation in accordance with the Declaration of Helsinki. Demographic and clinical characteristics of participants are shown in Table 1. This trial was registered on the Chinese Clinical Trial Registry (ChiCTR2000036959).

### 2.2. Electroacupuncture Stimulation

**Figure 2:**
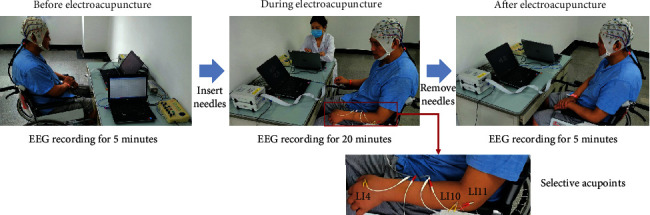
Details of experiment setup and three acupoints in the procedure of electroacupuncture.

The current study is based on our previous research [25]. The methods have been published in our previous study. An acupuncturist with more than 5 years of clinical experience inserted the sterile disposable acupuncture needles (Jiajian, 0.30 × 40 mm; Wuxi Jiajian Medical Instruments, Wuxi, China) in three acupoints (Hegu (LI4), Shousanli (LI10), Quchi (LI11)) [25]. These acupoints will be needled perpendicularly, with a depth of 10-15 mm approximately [25]. Following insertion, electrical stimulation parameters applied to the needles are intermittent wave, pulse signal with 250 *μ*s, sharp waveform, and frequency with 2 Hz [27, 28]. This electroacupuncture recipe is effective in activating the nerves and muscles. The current intensity was adjusted according to the patients' tolerance. After 20 minutes of retention of electroacupuncture, the needles were removed. Details of experiment setup and three acupoints in the procedure of electroacupuncture are shown in Figure 2.

### 2.3. EEG Recording

All patients' cortical activities were recorded by EEG in a sitting position with eyes opened before, during, and after electroacupuncture. EEG signals were recorded with BrainCap (Brain Products, Gilching, Germany) from 32-channel electrodes positioned according to the international 10-20 system at a sampling rate of 1000 Hz. The ground electrode and reference electrode were placed in the midsagittal line. All electrode impedances were maintained below 5 k*Ω*. The details of recording brain wave data were described in previous research [25].

### 2.4. EEG Processing

Data were performed with Matlab R2020a software (MathWorks, MA, USA) and using scripts based on the EEGLAB 2019 toolbox (http://sccn.ucsd.edu/eeglab/). To begin this process, raw data of the IO electrode was removed. Then, there were 31 channels (C3, C4, CP1, CP2, CP5, CP6, Cz, F3, F4, F7, F8, FC1, FC2, FC5, FC6, Fp1, Fp2, FT9, FT10, Fz, O1, O2, P3, P4, P7, P8, Pz, T7, T8, TP10, TP9) left for subsequent cortical network analysis. EEG signals were band-pass filtered into the beta-band (14–30 Hz), which play an important role in motor recovery [17, 29, 30]. Alpha-band- and beta-band-related brain functional indexes (e.g., coherence, multiscale permutation transfer entropy, oscillations) are most concerned in motor impairment and recovery after stroke. However, recent researches suggested that alpha-band is probably more associated with motor learning mechanisms and beta-band is associated with upper extremity motor recovery and neural networks that propagate activity between primary motor cortex and muscles [31, 32]. In this study, upper extremity motor recovery and the time since stroke onset are the main research purposes. Thus, beta-band was selected for EEG signal analysis. Then, EOG, ECG, and electroacupuncture-related artifacts were identified and eliminated through independent component analysis (ICA). ICA is a blind source separation algorithm that can be enabled to effectively separate eye movements and blink artifacts from EEG data. For each participant, 5 minutes before electroacupuncture, 20 minutes during electroacupuncture, and 5 minutes after needles were removed, artifact-free EEG signals were chosen for further PDC analysis.

Partial directed coherence (PDC) was used for effective connectivity calculation, which is a Granger causality-based measure in the frequency domain [23]. PDC is different from other undirected functional cortical networks based on phase synchronization (i.e., phase locking value and phase lag index). PDC measure the causal effects between channel signals, with direction. Briefly, the 31-channel EEG signals at a time point are defined as a vector

(1) $$\begin{array}{c} X\left(n\right)={\left[X_{1}\left(n\right),X_{2}\left(n\right),\;X_{3}\left(n\right),\cdots ,X_{N}\left(n\right)\right]}^{T}, \end{array}$$

where X_*N*_(*n*)(*N* = 1, 21, 2, ⋯, *N*) denote EEG signal from the *N*th channel. The EEG series can be modeled with a multivariate autoregressive (MVAR) model.

(2) $$\begin{array}{c} X\left(n\right)=\overset{p}{\underset{r=1}{\sum }}A_{r}X\left(n-r\right)+\text{W}\left(\text{n}\right), \end{array}$$

where *p* is the model order of MVAR, *A*_*r*_(*r* = 1, 2, ⋯, *p*) are the coefficient matrixes, and W(n) is a vector of multivariate uncorrelated white Gaussian noise. The model order *p* is defined by the Akaike Information Criterion (AIC) [33], and *A*_*r*_ are calculated by the Yule-Walker equation. Once *A*_*r*_ is estimated, *A*(*f*) could be calculated as

(3) $$\begin{array}{c} A\left(f\right)=I-\overset{p}{\underset{r=1}{\sum }}A_{r}e^{-2jfr\pi }, \end{array}$$

where *I* is the identity matrix. Then, the directed information flow from channel *j* to *i* at the frequency (PDC values) could be calculated as [23].

(4) $$\begin{array}{c} \text{ PDC}\left(i,j,f\right)=\frac{\;|\;A_{ij}\left(f\right)\;|\;}{\sqrt{\sum_{k}A_{kj}^{*}\left(f\right)A_{kj}\left(f\right)}}, \end{array}$$

where *A*_*ij*_(*f*) is the element of *A*(*f*) matrix and ∗ denotes the matrix transpose and complex conjugate. The PDC values range from 0 to 1; a higher value indicated a stronger information flow from channel *j* to *i*.

In this study, the PDC value that exceeded a threshold of 0.1 denotes significant causality, according to the spectral causality criterion (SCC) proposed by Schnider et al. [34]. PDC values for patients with infarct in the damaged left hemisphere (10 out of 18 in this study) were flipped along the midsagittal plane so that the right hemisphere corresponded to the ipsilesional hemisphere for all patients [35].

### 2.5. Network Analyses

In this study, the nodes of the network were 31-channel electrodes; 961 PDCs were estimated for all nodes in pair to build up the cortical network. The significant connection value (SCV) was considered to more than 0.1 according to SCC [23, 34]. The sum of PDCs (sPDC) of all SCV, the mean PDC (mPDC), and the number (*N*) of SCV were calculated to estimate the global connectivity of the cortical network [36].

### 2.6. Statistics

Statistical analyses were carried out using SPSS version 25.0 (IBM Inc., Chicago, IL, USA). Group differences of sPDC, mPDC, *N*, and the PDC value of each connection were tested with one-way ANOVA test. Two-tailed *t*-tests with Bonferroni adjustment were performed for all pairwise comparisons to reveal the changes before, during, and after electroacupuncture in each group. Significant level was set at 0.05 with a two-sided test.

## 3. Results

**Figure 3:**
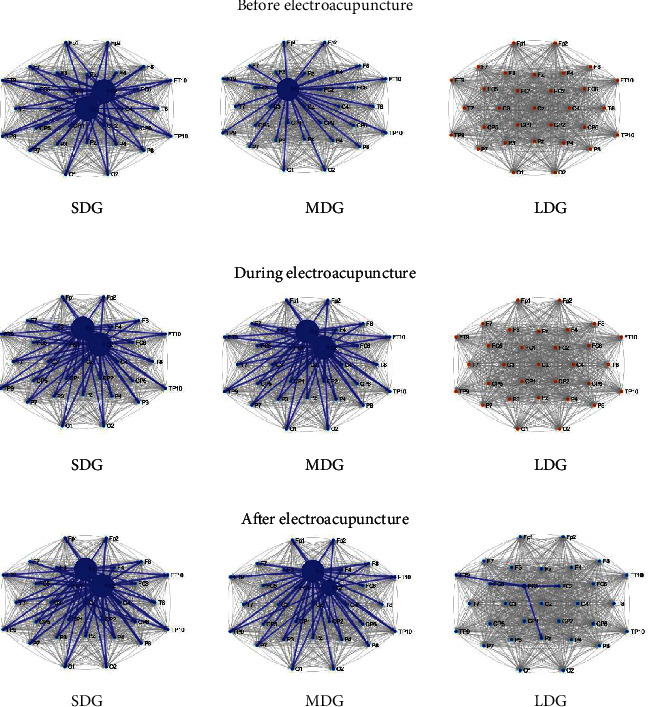
Grand averages of cortical networks at beta-band in patients with short duration, medium duration, and long duration of flaccid stage following stroke. Arrows indicate the significant connections. The size of the nodes is proportional to the degree.

The grand averages of cortical networks of three groups (SDG, MDG, and LDG) were representatively visualized with the Matlab R2020a software (Figure 3). Generally, the network of LDG presented a global less connectivity compared with SDG and MDG. Before electroacupuncture, two nodes were observed in the SDG patients' network, one node was observed in the MDG patients' network, and no nodes were observed in the LDG patients' network. During electroacupuncture, two nodes were observed in SDG and MDG patients' networks, and no nodes were observed in LDG patients' networks. After electroacupuncture, two nodes were observed in SDG and MDG patients' networks, and one node was observed in LDG patients' networks. The size of the nodes denotes the number of average SCV for patients. After electroacupuncture, larger nodes were observed in the SDG patients' network, and less node was observed in the LDG patients' network. That means patients with a duration of flaccid stage less than two months have more effective connectivity.

**Figure 4:**
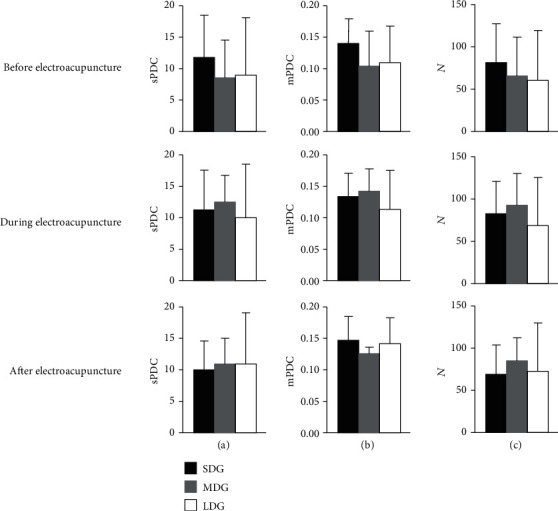
Global network connectivity at beta-band in patients with short duration, medium duration, and long duration of flaccid stage following stroke: (a) sum of PDCs (sPDC) of all significant connection values; (b) mean PDC (mPDC) of significant connections value; (c) number (*N*) of significant connection value.

Figure 4 presents the global network connectivity among the three groups before, during, and after electroacupuncture. There is no significant difference of sPDC, mPDC, and *N* among the three groups, indicating that the overall strength of the cortical connections and mean strength of SCV were not significantly different, no matter before, during, and after electroacupuncture.

**Figure 5:**
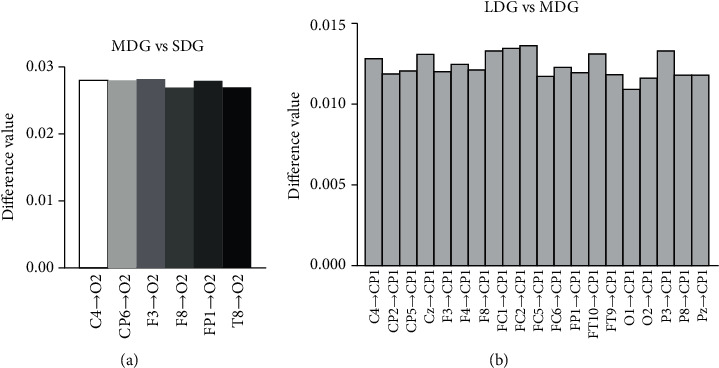
Significant difference of each connection between groups: (a) significant difference connections between medium duration and short duration of flaccid stage before electroacupuncture; (b) significant connections between long duration and medium duration of flaccid stage after electroacupuncture.

Figure 5 shows the detailed differences in each connection between groups. Six among 961 connections were significantly stronger for MDG compared with SDG (i.e., C4⟶O2, CP6⟶O2, F3⟶O2, F8⟶O2, Fp1⟶O2, T8⟶O2) before electroacupuncture (see details in Figure 5(a)). These results indicate that the direction information flow from channels C4, CP6, F3, F8, Fp1, and T8 to channel O2 is stronger in MDG than SDG before electroacupuncture. Twenty among 961 connections were significantly stronger for LDG compared with MDG (i.e., C4⟶CP1, CP2⟶CP1, CP5⟶CP1, Cz⟶CP1, F3⟶CP1, F4⟶CP1, F8⟶CP1, FC1⟶CP1, FC2⟶CP1, FC5⟶CP1, FC6⟶CP1, Fp1⟶CP1, FT10⟶CP1, FT9⟶CP1, O1⟶CP1, O2⟶CP1, P3⟶CP1, P4⟶CP1, P8⟶CP1, Pz⟶CP1) after electroacupuncture treatment (see details in Figure 5(b)). These results suggest that the direction information flow from channels C4, CP2, CP5, Cz, F3, F4, F8, FC1, FC2, FC5, FC6, Fp1, FT10, FT9, O1, O2, P3, P4, P8, and Pz to channel CP1 is stronger in LDG than MDG after electroacupuncture.

## 4. Discussion

The current study provides tentative cortical network evidence that the effective connectivity decreased at beta-band in patients with prolonged flaccid paralysis compared to those with short-duration and medium-duration flaccid stage. Patients with prolonged flaccidity seem to have no response to electroacupuncture treatment in topological configuration of the cortical network.

Disruption of cortical structural or functional networks after stroke has been broadly studied by neuroimaging or neural electrophysiological techniques [37, 38]. Prior study suggested an imbalance of intracortical inhibition between the affected and unaffected motor cortex and greater cortical density in the motor areas in patients with prolonged flaccidity [39]. Flaccidity has been verified associating with disruption of basal ganglia, internal and external capsule, and parietal lobe [7, 40]. In this study, 17 out of 18 stroke subjects were basal ganglia destruction subjects and 6 out of 18 subjects were parietal lobe destruction subjects, which is consistent with previous research findings.

Before electroacupuncture, we found that the effective connectivity is reduced at beta-band in patients with flaccidity for over six months compared to those with flaccidity for less than two months and those with flaccidity between two and six months. Effective connectivity of the medium-duration group is weak compared to that of the short-duration group. These findings suggested that functional connectivity turns into abnormalities or even disappearance along with the increasing duration of flaccid stage, which is in accordance with a major pattern of stroke patients: reduced cortical connectivity was correlated with clinical deficits [36].

During electroacupuncture, three interesting findings are (1) cortical network presented dynamic changes in topological configuration (the node goes from Cz to Fz) in SDG; (2) functional connectivity increased in MDG, and the connectivity transferred from contralesional into ipsilesional; and (3) network connectivity has no response to electroacupuncture in LDG. For SDG, the possible interpretation might be a reorganization occurred in the interhemispheres due to electroacupuncture. Recent studies have verified that cortical reorganization may contribute to the recovery of motor function after stroke [41]. For MDG, electroacupuncture may reduce inhibition of the affected hemisphere by the unaffected hemisphere, resulting in increased connectivity of the affected hemisphere. As we know, the major model of functional recovery is the interhemispheric competition model. The model hypothesized that a mutual, balanced inhibition between the hemispheres was disrupted following stroke, resulting in decreased inhibition of the unaffected hemisphere by the affected hemisphere and increased inhibition of the affected hemisphere by the unaffected hemisphere [14]. For LDG, electroacupuncture may not arouse the changes of the cortical network. This finding is consistent with that of Daly et al. who found that the patient will probably not regain any function in response to treatment if he had flaccid paralysis for more than one year after stroke [6].

After electroacupuncture, we found that the size of nodes reduced in MDG, suggesting that the dynamic changes of the cortical network due to electroacupuncture maintain for a while after treatment. The size of node is unchanged in SDG, indicating that the neuroplasticity in patients with different durations of flaccid stage is different.

The sPDC, mPDC, and *N* of SCV are the global network connectivity measurements. There is no significant difference among the three groups before, during, and after electroacupuncture. A possible interpretation is that the disruptions of global network connectivity are similar among the three groups. The PDC value of each connection was also calculated in this study. Comparing to SDG, the stronger information flow from six channels (i.e., C4, CP6, F3, F8, Fp1, and T8) to channel O2 was observed in MDG before electroacupuncture. After electroacupuncture, the stronger information flow from twenty channels (e.g., C4, CP2, CP5, Cz, F3) to channel CP1 was observed in LDG comparing to MDG. However, these connections were uninvolved in the significant connection value (see Figure 2). Thus, we speculated that these connections are not important for global network connectivity analysis in this study.

Our study suggested that patients with different durations of flaccid stage have various responses to electroacupuncture in the topological configuration of the cortical network. These findings.

may help us to understand that the same electroacupuncture prescriptions are not applicable to all stroke patients with flaccid paralysis. The customization of electroacupuncture therapeutic regimen according to flaccid stage is needed. However, which electroacupuncture regimens are more beneficial to patients is unclear and needs to be explored in future research. There are several limitations in this study. Firstly, electroacupuncture was performed only once on each stroke patient; the clinical significance of electroacupuncture therapy for stroke patients is hardly explained in this study. Secondly, we did not set up a control group with three randomized acupoints or three nonacupoints so that the results are not that convincing. Thirdly, the repeatability of these results may be affected due to the cross-sectional trail design. Therefore, the long-term electroacupuncture therapy with a large sample size would be designed and implemented to explore the time window and the prescription of electroacupuncture on stroke patients in the future.

## 5. Conclusions

This study investigated the cortical network changes from different durations of flaccid stage in stroke patients during electroacupuncture. Our results revealed that the cortical connectivity weakened and less responded to electroacupuncture in patients with prolonged flaccid stage. These findings may be helpful for modulating the formulation of electroacupuncture treatment in motor recovery after stroke.

## Figures and Tables

**Table 1:** Demographic and clinical data of participants.

Group-ID	Gender	Age (years)	Site of lesion	Ischemia/hemorrhage	Days since stroke	Side of lesion	Side of hemiparesis	Handedness	NIHSS	BI
SDG-1	M	70	BG, FL PL, pons	I	55	R	L	R	11	40
SDG-2	M	60	BG, CR	I	15	R	L	R	11	60
SDG-3	M	56	BG	H	22	L	R	R	13	40
SDG-4	M	64	BG, CR, FL	I	17	L	R	R	12	25
SDG-5	F	71	BG, CO, FL, PL, pons	I	11	R	L	R	7	20
SDG-6	F	77	BG, CR, FL, PL	I	25	R	L	R	8	40
Mean (±SD)		66.33 (±7.09)			24.17 (±14.52)				10.33 (±2.13)	37.50 (±12.83)
MDG-1	M	77	BG, FL, PL	I	98	R	L	R	9	50
MDG-2	M	60	BG	H	108	R	L	R	4	55
MDG-3	M	60	BG, CO, FL, OL, TL	I	62	L	R	R	14	30
MDG-4	M	61	Th, OL, TL	I	111	L	R	R	9	65
MDG-5	M	60	BG, IC	I	146	L	R	R	8	40
MDG-6	F	59	TL	I	118	L	R	R	15	30
Mean (±SD)		62.83 (±6.36)			107.17 (±25.05)				9.83 (±3.72)	45.00 (±12.91)
LDG-1	M	66	BG	I	938	R	L	R	5	50
LDG-2	M	62	BG, FL, PL	I	198	L	R	R	10	70
LDG-3	M	48	BG, FL, PL, TL	I	407	L	R	R	10	65
LDG-4	M	44	BG	H	185	L	R	R	7	70
LDG-5	F	68	BG	H	1184	R	L	R	29	10
LDG-6	F	75	BG, Th	I	370	L	R	R	24	10
Mean (±SD)		60.50 (±11.01)			547.00 (±379.13)				14.17 (±9.01)	45.83 (±26.21)

Abbreviations: BI: Barthel Index; BG: basal ganglia; CO: centrum ovale; CR: corona radiate; F: female; FL: frontal lobe; H: hemorrhage; I: ischemia; IC: internal capsule; L: left; M: male; NIHSS: National Institutes of Health Stroke Scale; OL: occipital lobe; PL: parietal lobe; R: right; Th: thalamus; TL: temporal lobe.

## Data Availability

The data used to support the findings of this research are available from the corresponding author.
